# Effectiveness of an Interactive mHealth App (EVITE) in Improving Lifestyle After a Coronary Event: Randomized Controlled Trial

**DOI:** 10.2196/48756

**Published:** 2024-04-22

**Authors:** María Ángeles Bernal-Jiménez, German Calle, Alejandro Gutiérrez Barrios, Livia Luciana Gheorghe, Celia Cruz-Cobo, Nuria Trujillo-Garrido, Amelia Rodríguez-Martín, Josep A Tur, Rafael Vázquez-García, María José Santi-Cano

**Affiliations:** 1 Faculty of Nursing and Physiotherapy, University of Cádiz Cádiz Spain; 2 Institute of Biomedical Research and Innovation of Cádiz Cádiz Spain; 3 Research Group on Nutrition: Molecular, Pathophysiological and Social Issues, University of Cádiz Cádiz Spain; 4 Cardiology Unit, Puerta del Mar Hospital Cádiz Spain; 5 Biomedicine, Biotechnology and Public Health Department, University of Cádiz Cádiz Spain; 6 Research Group on Community Nutrition & Oxidative Stress, University of the Balearic Islands Palma de Mallorca Spain; 7 Network Biomedical Research Center "Pathophysiology of Obesity and Nutrition" Carlos III Health Institute Madrid Spain

**Keywords:** coronary artery disease, healthy lifestyle, mHealth, mobile health, percutaneous coronary intervention, randomized controlled trial, secondary prevention, therapeutic adherence

## Abstract

**Background:**

Coronary heart disease is one of the leading causes of mortality worldwide. Secondary prevention is essential, as it reduces the risk of further coronary events. Mobile health (mHealth) technology could become a useful tool to improve lifestyles.

**Objective:**

This study aimed to evaluate the effect of an mHealth intervention on people with coronary heart disease who received percutaneous coronary intervention. Improvements in lifestyle regarding diet, physical activity, and smoking; level of knowledge of a healthy lifestyle and the control of cardiovascular risk factors (CVRFs); and therapeutic adherence and quality of life were analyzed.

**Methods:**

This was a randomized controlled trial with a parallel group design assigned 1:1 to either an intervention involving a smartphone app (mHealth group) or to standard health care (control group). The app was used for setting aims, the self-monitoring of lifestyle and CVRFs using measurements and records, educating people with access to information on their screens about healthy lifestyles and adhering to treatment, and giving motivation through feedback about achievements and aspects to improve. Both groups were assessed after 9 months. The primary outcome variables were adherence to the Mediterranean diet, frequency of food consumed, patient-reported physical activity, smoking, knowledge of healthy lifestyles and the control of CVRFs, adherence to treatment, quality of life, well-being, and satisfaction.

**Results:**

The study analyzed 128 patients, 67 in the mHealth group and 61 in the control group; most were male (92/128, 71.9%), with a mean age of 59.49 (SD 8.97) years. Significant improvements were observed in the mHealth group compared with the control group regarding adherence to the Mediterranean diet (mean 11.83, SD 1.74 points vs mean 10.14, SD 2.02 points; *P*<.001), frequency of food consumption, patient-reported physical activity (mean 619.14, SD 318.21 min/week vs mean 471.70, SD 261.43 min/week; *P*=.007), giving up smoking (25/67, 75% vs 11/61, 42%; *P*=.01), level of knowledge of healthy lifestyles and the control of CVRFs (mean 118.70, SD 2.65 points vs mean 111.25, SD 9.05 points; *P*<.001), and the physical component of the quality of life 12-item Short Form survey (SF-12; mean 45.80, SD 10.79 points vs mean 41.40, SD 10.78 points; *P*=.02). Overall satisfaction was higher in the mHealth group (mean 48.22, SD 3.89 vs mean 46.00, SD 4.82 points; *P*=.002) and app satisfaction and usability were high (mean 44.38, SD 6.18 out of 50 points and mean 95.22, SD 7.37 out of 100).

**Conclusions:**

The EVITE app was effective in improving the lifestyle of patients in terms of adherence to the Mediterranean diet, frequency of healthy food consumption, physical activity, giving up smoking, knowledge of healthy lifestyles and controlling CVRFs, quality of life, and overall satisfaction. The app satisfaction and usability were excellent.

**Trial Registration:**

Clinicaltrials.gov NCT04118504; https://clinicaltrials.gov/study/NCT04118504

## Introduction

Ischemic heart disease, or coronary artery disease (CAD), remains one of the leading causes of mortality worldwide, accounting for 16% of all deaths [1,2]. Coronary revascularization using percutaneous coronary intervention (PCI) is the main treatment option in patients with CAD as it restores coronary blood flow and has excellent clinical outcomes [3]. This procedure, however, does not prevent either the formation of further obstructions in the coronary arteries treated or the disease from progressing with the appearance of new lesions in other parts of the coronary tree. Therefore, secondary prevention strategies aimed at achieving a healthy lifestyle and the control of cardiovascular risk factors (CVRFs) are essential [4].

Secondary prevention and cardiac rehabilitation programs can improve lifestyle, therapeutic adherence, and the control of CVRFs. This facilitates a decrease in the risk of a further cardiovascular event that requires another PCI or even surgery, leading to a reduction in health care costs [5,6]. However, a large number of patients do not make these recommendations a part of their everyday lifestyle or do not take their medication correctly after experiencing a cardiovascular event, which stops them from achieving the treatment goals established [4,7]. The data from the EUROASPIRE IV survey conducted in Europe demonstrated that only 51% of patients who had experienced a coronary event were advised to participate in a cardiac rehabilitation program, and of those, only 41% took part [7]. This suggests that the implementation of attendance-based secondary prevention programs after acute coronary syndrome has stagnated somewhat.

Moreover, during the COVID-19 pandemic, secondary prevention and cardiac rehabilitation programs were affected and even suspended, thereby seriously limiting patient access to available health services [8,9]. Thus, it is necessary to implement innovative secondary prevention strategies that improve adherence to a healthy lifestyle and the long-term treatment prescribed.

The use of information and communication technology may facilitate new communication pathways between patients and health professionals and allow patients more autonomy in the control of their disease while providing professionals with more information about their patients’ health status. These days, over 57% of homes have access to the internet, and over 75% of the world’s population has a mobile phone with internet access [10,11]. These data highlight that these technologies and smartphones should form part of the communication between patients and health care professionals, as they can be valuable tools for controlling health and lifestyle. The beneficial effects of mobile health (mHealth) systems have been observed in primary prevention patients at high risk of cardiovascular disease [12-15]. A recent systematic review with 2250 participants that analyzed the results obtained after implementing cardiac telerehabilitation programs concluded that these strategies are a way to improve the use and reach of cardiac rehabilitation programs [16]. Likewise, recent meta-analyses of clinical trials performed in patients with CAD concluded that secondary prevention programs involving telehealth interventions may be useful either in isolation or combined with on-site cardiac rehabilitation and that they are related to a decrease in adverse events, lifestyle improvements, and better control of CVRFs [17,18]. These studies reflect the need for further similar research to evaluate the effectiveness of telehealth interventions in this context.

This clinical trial aimed to evaluate the effect of an mHealth intervention on people with heart disease who had undergone PCI in terms of the following variables: improvements in lifestyle, namely diet, physical activity (PA), and smoking; level of knowledge of a healthy lifestyle and the control of CVRFs; and therapeutic adherence and quality of life.

## Methods

### Study Design

A statistician-blinded randomized controlled clinical trial with a parallel group design was conducted in patients with CAD who underwent a PCI with stent implantation in the cardiology unit of a public specialty reference hospital in the province of Cádiz, Spain, between November 2019 and June 2022.

### Study Sample

A total of 134 patients participated, 67 in the intervention group and 67 in the control group. The initial sample size proposed was 240 participants, but the increase in hospital occupancy due to the COVID-19 pandemic reduced the number of patients scheduled for coronary intervention with a nonurgent pathology. However, this sample size is sufficient to detect an effect size (Cohen *d*) of 0.5 on adherence to the Mediterranean diet (mean 8.7, SD 2 points) [19], time spent doing PA (mean 416.8, SD 374 min/week) [20], level of knowledge of CVRFs (mean 104.7, SD 9.7) [21], and a difference in the prevalence of stopping smoking of over 20% [22], with a 95% CI and statistical power of 90%.

### Eligibility Criteria

The study included patients with CAD aged between 18 and 75 years who owned a smartphone with an internet connection during the whole study period.

Those excluded from the study were people with severe heart failure, congenital, structural, or serious rheumatic heart disease, chronic kidney or liver disease, physical disability or dementia, and those who were already using a health control app or whose mobile phone was incompatible with the app.

### Recruitment, Randomization, and Masking

#### Overview

The patients meeting the inclusion criteria were randomized and assigned to either the mHealth group or the control group receiving standard health care using a computerized random number generator 1:1. The randomization sequence was generated by staff at the study’s coordination center at the university who were not involved in recruiting the participants. The coordinators did not know the randomization group. The researchers analyzing the results were blinded to the allocation of participants. Blinding the participants was not possible because of the nature of the intervention.

Eligible patients and their caregivers were approached during their hospital admission, after the PCI, and before discharge. All the patients were informed of the characteristics of the project and were invited to volunteer to participate and sign the informed consent form. The patients who agreed to participate underwent an initial assessment and were given a follow-up appointment 9 months later. The patients were given an email address and telephone number through which they could resolve any queries or doubts during the study period. Before their hospital discharge, all the patients were encouraged to lead healthy lifestyles. Moreover, they were given written information about CVRFs, lifestyle objectives, examples of healthy diets, and recommendations about the daily portions of each food group.

#### Control Group

The patients in the standard health care group received advice about medication and lifestyle, the Mediterranean diet, PA, stopping smoking, and therapeutic adherence. They were also provided with written recommendations.

#### mHealth Group

The mHealth intervention began during their hospital stay, after the coronary event. All the patients in this group had an app installed on their mobile phone or tablet and completed a brief online tutorial to learn how to use it. The participants were advised to use the app for a minimum of 15 minutes daily during the 36-week follow-up period. The app established aims to achieve self-control of PA, food consumption, blood pressure, smoking, and therapeutic adherence. The app is based on the phases of change theory (attention, retention, memory, action, and motivation) [23] and on making the process pleasing [24,25]. The user’s attention was caught through warnings and bright, attractive colors on the user interface; retention was encouraged by reminders, repetition, and graphs; instructions, advice, and feedback prompted action; and motivation to change was boosted by internal comparisons (progress graphs), setting goals, self-monitoring, and feedback.

**Figure 1:**
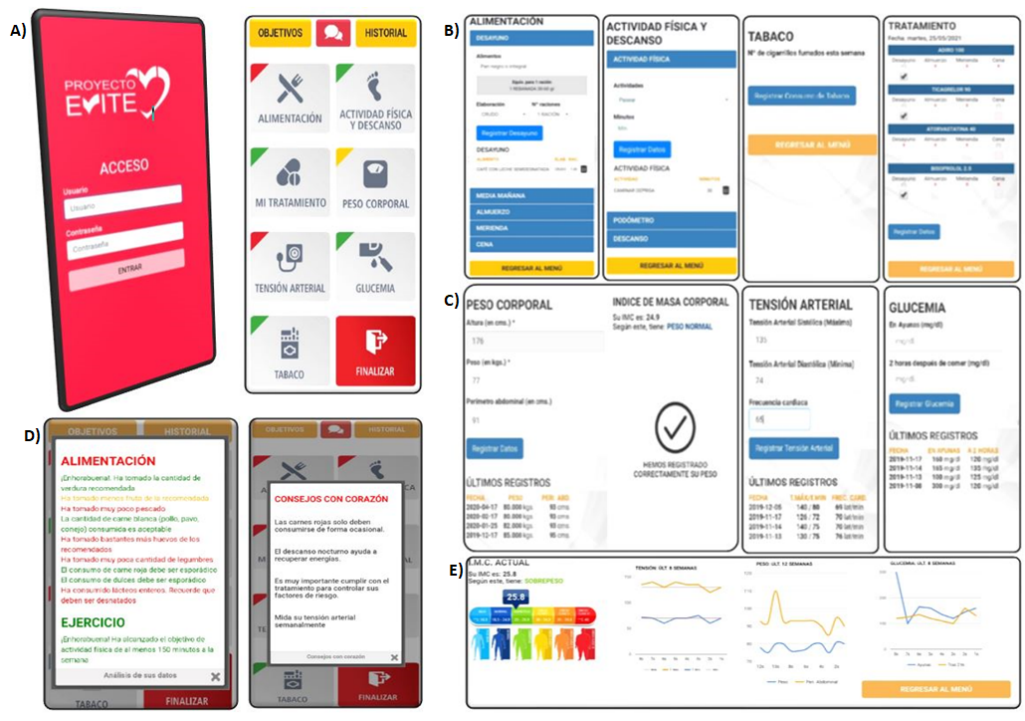
(A) Start page app of the EVITE project. (B) Diet, physical activity, smoking cessation, and treatment in the top row (from left to right). (C) Body weight, BMI, blood pressure, and capillary blood glucose modules in the middle row (from left to right). (D) Weekly feedback traffic light and messages, automatic text message reminders. (E) Record: graphics of evolution and progress of BMI, blood pressure, body weight and waist circumference (WC), and capillary blood glucose in the bottom row (from left to right).

A nurse resolved any queries the participants had through the messaging function integrated into the app. The technical data about the website, app, and its contents and components were described previously in greater detail [26]. Briefly, the app provides (1) education about following a healthy lifestyle and about the recommended therapeutic goals in the clinical practice guidelines regarding food, PA, body weight, blood pressure, blood sugar, stopping smoking, and adhering to treatment. The app provided the participants with information on their screens to help them plan a healthier lifestyle and adhere to their treatment; (2) self-monitoring, using records, and a self-checking function to allow patients to monitor each behavioral goal about nutrition, PA, tobacco consumption, blood pressure, body weight, capillary blood glucose in patients with diabetes mellitus and treatment adherence; and (3) motivation to improve and maintain lifestyle habits through automatic reminders about healthy habits generated randomly in a pop-up screen and through personalized messages about reaching goals related with improving their lifestyle and treatment adherence, and recommendations about aspects to be improved (Figure 1).

The personalized messages are produced in response to the information recorded by the patient over the previous 7 days. The patients received weekly feedback through pop-ups that appeared when opening the app. The messages appeared as short sentences in green (when the set goals had been reached), yellow (if they had partially been reached), and red (when the goal was pending), depending on the degree of compliance and control of the goals. As a reminder of achievements and aspects to improve, the top left corner of each section of the app’s main screen appeared in the color corresponding to the goals reached during the previous week. The participants could follow their evolution and progress through the graphics generated from the information they had recorded over the previous 8 weeks (Figure 1).

Through its different components (website, messages, emails, and calls), participants were encouraged to (1) follow a healthy eating pattern based on the Mediterranean diet; (2) perform PA of duration and intensity in line with the recommendations of their cardiologist; (3) stop smoking; (4) monitor their blood pressure; and (5) adhere to their treatment by associating taking medication with daily activities, establishing set times for taking it, and with support from a relative, etc. We hypothesize that information is the first step to following a healthy lifestyle. The self-monitoring and recording in the app improves the patients’ awareness of their lifestyle behavior, and motivation promotes the initiation and continuation of changes in behavior over time.

### Follow-Up

All the participants in both the mHealth group and the control group were evaluated after 36 weeks. Their digital medical records were reviewed to check and record clinical data of medical visits, recent analyses (the same data from the baseline plasma analysis), and adverse events that occurred over that period. Due to the COVID-19 pandemic, the questionnaires were administered by telephone.

### Outcome Variables

#### Overview

The primary outcome variables were adherence to the Mediterranean diet, frequency of consumption of foods, PA performed, smoking (giving up and nicotine dependence test), level of knowledge of a healthy lifestyle, and the control of CVRFs, therapeutic adherence, quality of life, well-being, and satisfaction.

The secondary outcome variables were BMI, WC, systolic blood pressure (SBP), diastolic blood pressure (DBP), heart rate, glycated hemoglobin (HbA_1c_), total cholesterol, high-density lipoprotein (HDL) cholesterol, low-density lipoprotein (LDL) cholesterol, triglycerides, emotional status, major adverse cardiovascular events (MACEs), and other complications.

While the patients were hospitalized and before their discharge, measurements were taken of their weight and height to calculate their BMI, WC, SBP, and DBP following standard clinical methods. Moreover, records were made of sociodemographic variables (age, sex, and educational level), history of CVRFs (diabetes mellitus, arterial hypertension, hyperlipidemia, obesity, and tobacco consumption), and history of cardiovascular disease (infarction, angina, and stroke). Furthermore, the patient’s digital medical records were used to obtain data about left ventricular ejection fraction [27] and plasma analysis (total cholesterol, LDL cholesterol, HDL cholesterol, baseline glycemia, HbA_1c_, creatinine, and C-reactive protein).

#### Primary Outcomes

##### Smoking

Nicotine dependence was assessed using the Fagerström test [28], with scores below 4 points considered low dependence, scores between 4 and 7 considered moderate dependence, and scores from 8 to 10 considered points high dependence. Stopping smoking was self-reported.

##### Adherence to the Mediterranean Diet and Frequency of Food Consumption

To assess food intake, the following surveys were used: (1) the Mediterranean Adherence Score (maximum score of 14 points; scores below 9 are classified as low adherence, and scores above 9 are classified as high adherence) [19,29]; and (2) the food frequency questionnaire [30].

##### Physical Activity

Moreover, the time spent performing PA was assessed using the Minnesota Physical Activity Survey (min/week) [31,32], which includes detailed instructions and a list of defined activities. Participants are asked to mark those performed during the past year, month, and week (the activities are expressed in the number of days per week and minutes per day). Total energy expenditure from leisure time PA can be calculated with this questionnaire depending on the type performed: light (≤4 metabolic equivalents [METs]), moderate (4.5-5.5 METs), or heavy (≥6 METs). For this research, only the information referring to the past week was used as the min/week of light PA. The reason was that the participants only performed light PA after PCI. Furthermore, this allowed the comparison of results with other studies that expressed PA in min/week and with the recommendations of the cardiovascular disease prevention guidelines [4]. The PA in the household was omitted. Therapeutic adherence was assessed using the 4-item Morisky Green and Levine Medication Adherence Questionnaire (MGL MAQ). This was considered good if the 4 questions were answered correctly (NO/YES/NO/NO) [33,34].

##### Level of Knowledge of CVRFs and a Healthy Lifestyle

The level of knowledge of CVRFs and a healthy lifestyle was examined using a validated scale [21]. The scale consists of 24 items with 5 response options and a maximum score of 120 points. Each item is scored from 1 to 5 points, with the highest score corresponding to the most correct answer. The patient’s level of knowledge was considered high when they responded correctly to over 75% of the items (90 points).

##### Quality of Life and Well-Being

The physical and mental dimensions of quality of life were assessed by the SF-12 survey [35], in which the highest scores imply better health-related quality of life. The possible answers for each item are presented as a Likert-type scale, with the number of options varying from 3 to 6 points, depending on the item. To calculate each dimension, the items were recoded, aggregated, and transformed into a score ranging from 0 (worse health status) to 100 points (better health status) [36,37]. Well-being was assessed using the World Health Organization-5 Well-Being Index questionnaire, on which the lowest score is 0 points and the highest 100, a higher score indicating greater well-being [38].

##### Patient Overall Satisfaction

Overall satisfaction with the health care received was assessed using a specific questionnaire developed by the research team. The questionnaire consists of 10 questions with 5 response options scored from 1 to 5, from the lowest to the highest level of satisfaction. The maximum score is 50 points, with higher scores corresponding to more satisfaction.

#### Secondary Outcomes

##### BMI, WC, SBP, DBP, Heart Rate, HbA1c, and Lipids

BMI, WC, SBP, DBP, heart rate, HbA_1c_, total cholesterol, HDL cholesterol, LDL cholesterol, and triglycerides were obtained following the standard methods mentioned above.

##### Anxiety and Depression

Levels of anxiety and depression were analyzed using the questionnaire developed by Goldberg et al [39]. This questionnaire consists of 2 subscales; a score over 4 on the anxiety subscale is considered a diagnosis of anxiety, and a score over 2 on the depression subscale is considered a diagnosis of this disorder. The maximum score on each subscale is 9 points, with high scores reflecting a more serious problem [40].

##### MACEs and Other Complications

At the end of the follow-up period, each patient’s digital medical records were analyzed for MACEs (cardiovascular death, acute myocardial infarction, stent thrombosis, new revascularization of target injury, stroke, and other possible complications that can occur, such as bleeding, angina, and the revascularization of another vessel) that had occurred.

##### Patient App Satisfaction and Usability

Satisfaction with the app itself was assessed using a specific questionnaire developed by the research team. The questionnaire consists of 10 questions with 5 response options scored from 1 to 5, from the lowest to the highest level of satisfaction. The maximum score is 50 points, with higher scores corresponding to more satisfaction.

The app’s usability was assessed in the intervention group using the System Usability Scale (SUS) questionnaire, which evaluates user acceptance, with scores ranging from 0 to 100 points [41]. A higher score is an indication of better usability. The scores are classified as excellent for >80.3, good for 68-80.3, poor for 51-67, and very poor for <51.

### Statistical Analysis

A descriptive statistical analysis was performed (mean, median, SD, 95% CI, interquartile interval, frequencies, and percentages). The primary quantitative results from the 2 groups—mHealth and control—were compared using the Student 2 tailed *t* test (variables with normal distribution) and the Mann-Whitney *U* test (variables not distributed normally). The *χ*^2^ or Fisher test was used for the comparison of proportions. A 2-tailed *P* value of <.05 was considered statistically significant. The data analysis was performed using SPSS (version 24.0; IBM Corp) for Windows.

### Ethical Considerations

The Biomedical Research Ethics Committee of Andalusia approved the study (003_ene19_PI-EVITE-18). The patients signed a written informed consent form. The app included data encryption mechanisms and guaranteed safety measures in accordance with current European Data Protection Regulations. This study was registered on ClinicalTrials.gov (NCT04118504).

## Results

### Overview

**Figure 2:**
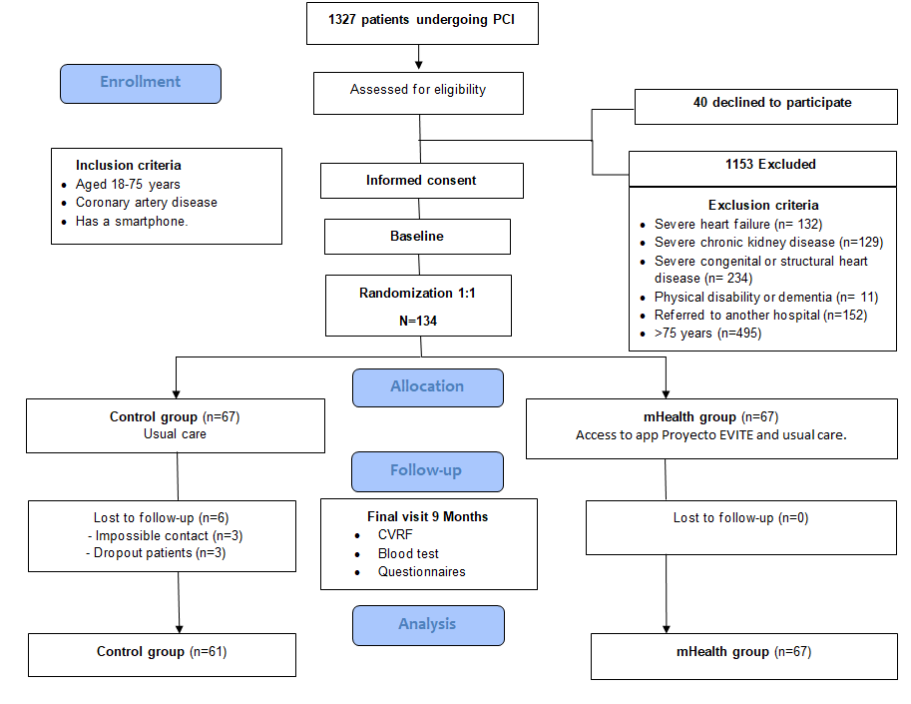
Flow diagram of the study.

Figure 2 shows a flowchart of the process for recruiting the participants to the clinical trial. During the recruitment period, 1327 patients underwent a PCI and were evaluated for inclusion in the study. After applying the inclusion and exclusion criteria, 1153 were excluded, and 40 refused to participate. The 134 remaining patients were randomized: 67 to the control group and 67 to the mHealth group. There were 6 dropouts in the control group during the follow-up. No participants dropped out of the intervention group. In the end, 128 were analyzed: 61 from the control group and 67 from the mHealth group.

Table 1 shows the baseline characteristics of the participants. Most were male (92/128, 71.9%) with a mean age of 59.49 (SD 8.97) years. In general, both groups were homogeneous.

**Table 1:** Patient baseline characteristics.

		Total (n=128)	mHealth (n=67)	Control (n=61)	*P* value
Male, n (%)		92 (71.9)	53 (79)	39 (64)	.05
**Age (years)**					.01
	Mean (SD)	59.49 (8.97)	57.70 (8.167)	61.46 (9.476)	
	95% CI	57.92-61.06	55.71-59.69	59.03-63.89	
**Educational level, n (%)**					.17
	No studies	1 (1.3)	0 (0)	1 (2)	
	Primary	21 (26.9)	6 (17)	15 (36)	
	Middle school	37 (47.7)	19 (53)	18 (43)	
	High school	19 (24.4)	11 (31)	8 (19)	
**BMI (kg/m^2^)**					.55
	Mean (SD)	29.34 (4.99)	29.09 (5.02)	29.61 (4.99)	
	95% CI	28.47-30.21	27.86-30.32	28.34-30.89	
**Waist circumference (cm)**					.52
	Mean (SD)	104.15 (13.78)	104.91 (10.75)	103.31 (16.55)	
	95% CI	101.69-106.60	102.24-107.57	98.99-107.62	
Overweight, n (%)		50 (39.1)	28 (42)	22 (36)	.29
Obesity, n (%)		54 (42.2)	25 (37)	29 (48)	.29
Hypertension, n (%)		74 (57.8)	38 (57)	36 (59)	.79
Diabetes mellitus, n (%)		39 (30.5)	13 (19)	26 (43)	.004
Dyslipidemia, n (%)		66 (51.6)	31 (46)	35 (57)	.20
Smoking, n (%)		59 (46.1)	33 (49)	26 (43)	.45
Former smoker, n (%)		35 (27.3)	21 (31)	14 (23)	.28
Morbidities^a^, n (%)		8 (6.3)	3 (5)	5 (8)	.42
**LVEF^b^ (%)**					.78
	Mean (SD)	58.27 (9.08)	58.04 (8.71)	58.56 (9.62)	
	95% CI	56.40-60.14	55.61-60.46	55.52-61.60	
**Reason for catheterization, n (%)**					.04
	Stable angina	32 (25.0)	11 (16)	21 (34)	
	Unstable angina	19 (14.8)	8 (12)	11 (18)	
	NSTEMI^c^	29 (22.7)	19 (28)	10 (16)	
	STEMI^d^	48 (37.5)	29 (43)	19 (31)	
**Number of implanted stents**					.63
	Mean (SD)	2.34 (1.65)	2.40 (1.89)	2.26 (1.36)	
	95% CI	2.05-2.6	1.94-2.86	1.91-2.61	
**Discharge treatment, n (%)**					
	Anticoagulants	6 (4.7)	3 (5)	3 (5)	.93
	Antiplatelet	126 (98.4)	66 (100)	60 (97)	.14
	Antihypertensives	124 (96.9)	65 (99)	59 (95)	.28
	Nitrates	14 (10.9)	4 (6)	10 (16)	.06
	Insulin	7 (5.5)	3 (5)	4 (7)	.63
	Oral antidiabetics	41 (32.0)	16 (24)	25 (40)	.05
	Statins	120 (93.8)	64 (97)	56 (30)	.12

^a^Chronic obstructive pulmonary disease, kidney disease, and obstructive sleep apnea syndrome.

^b^LVEF: left ventricular ejection fraction.

^c^NSTEMI: non–ST-segment elevation myocardial infarction.

^d^STEMI: ST-segment elevation acute myocardial infarction.

### Primary Outcomes

#### Overview

The outcome variables at the end of the 9-month follow-up period are shown in Table 2.

**Table 2:** Primary outcome variables at baseline and 9 months.

					Total (n=128)		mHealth (n=67)		Control (n=61)		*P* value
**Mediterranean diet**											
	**Mediterranean adherence score**										
		**Baseline**									.57^a^
			Mean (SD)	7.38 (2.28)		7.24 (2.21)		7.52 (2.37)			
			95% CI	6.98-7.77		6.70-7.78		6.92-8.13			
		**9 months**									<.001^a^
			Mean (SD)	11.03 (2.05)		11.83 (1.74)		10.14 (2.02)			
			95% CI	10.66-11.41		11.39-12.27		9.60-10.68			
	**Good adherence, n (%)**										
		Baseline		36 (28.1)		18 (27)		18 (30)		.74	
		9 months		99 (83.2)		57 (91)		42 (75)		.02	
**Food consumption, n (%)**											
	**Red meat ≤1/week**										
		Baseline		54 (42.2)		32 (48)		22 (36)		.05	
		9 months		104 (86.7)		60 (95)		44 (77)		.001	
	**Vegetables ≥2/day**										
		Baseline		36 (28.1)		15 (22)		21 (34)		.54	
		9 months		76 (62.8)		48 (75)		28 (49)		.03	
	**Fruits ≥2/day**										
		Baseline		48 (37.5)		23 (34)		25 (41)		.81	
		9 months		97 (80.1)		58 (91)		39 (68)		.02	
	**Whole grains ≥1/day**										
		Baseline		9 (7.1)		7 (11)		2 (3)		.48	
		9 months		61 (50.4)		41 (64)		20 (35)		.003	
	**Industrial pastry <2/week**										
		Baseline		57 (44.6)		29 (43)		28 (46)		.47	
		9 months		112 (92.6)		60 (94)		52 (91)		.003	
**Physical activity** (**patient-reported)**											
	**Minnesota** **Physical Activity Survey (min/week)**										
		**Baseline**									.51
			Mean (SD)	368.97 (304.06)		385.76 (286.53)		350.19 (323.99)			
			95% CI	315.14-422.80		315.32-456.20		265.75-434.62			
		**9 months**									.007
			Mean (SD)	549.76 (300.87)		619.14 (318.21)		471.70 (261.43)			
			95% CI	495.14-604.37		539.00-699.28		401.68-541.71			
**Smoking status, n(%)**											
	**Smoker**										
		9 months		23 (18.0)		8 (12)		15 (25)		.07	
	Smoking cessation				36 (61.0)		25 (76)		11 (42)		.01
	**Nicotine dependence (** **Fagerström test;** **score)**										
		**Baseline**									.58
			Mean (SD)	4.91 (2.57)		4.74 (2.45)		5.10 (2.73)			
			95% CI	4.28-5.54		3.90-5.59		4.09-6.10			
		**9 months**									.02^a^
			Mean (SD)	1.56 (2.61)		0.93 (2.29)		2.27 (2.82)			
			95% CI	0.86-2.27		0.06-1.81		1.14-3.40			
**Cardiovascular risk factors**											
	**CVRF^b^ knowledge (score)**										
		**Baseline**									.003
			Mean (SD)	104.98 (9.83)		107.42 (8.90)		102.31 (10.17)			
			95% CI	103.26-106.70		105.25-109.70		99.71-104.92			
		**9 months**									<.001^a^
			Mean (SD)	115.19 (7.47)		118.70 (2.65)		111.25 (9.05)			
			95% CI	113.84-116.55		118.03-119.37		108.83-113.67			
**Medication adherence, n (%)**											
	**MGL MAQ^c^**										
		Baseline		53 (50.0)		26 (47)		27 (53)		.56	
		Final		98 (81.7)		54 (84)		44 (79)		.4	
**Quality of life**											
	**SF-12^d^ PCS^e^ (score)**										
		**Baseline**									.20
			Mean (SD)	39.55 (12.24)		40.87 (11.98)		38.10 (12.46)			
			95% CI	37.39-41.71		37.93-43.82		34.87-41.32			
		**9 months**									.03^a^
			Mean (SD)	43.73 (10.96)		45.80 (10.79)		41.40 (10.78)			
			95% CI	41.74-45.72		43.08-48.51		38.52-44.29			
	**SF-12 MCS^f^ (score)**										
		**Baseline**									.02
			Mean (SD)	48.61 (10.21)		50.51 (9.00)		46.52 (11.09)			
			95% CI	46.81-50.41		48.30-52.72		43.65-49.38			
		**9 months**									.57^a^
			Mean (SD)	52.01 (9.54)		52.40 (9.27)		51.58 (9.89)			
			95% CI	50.28-53.75		50.06-54.73		48.93-54.23			
**Well-being**											
	**WHO-5^g^ (score)**										
		**Baseline**									.24
			Mean (SD)	60.88 (19.49)		62.81 (18.95)		58.73 (20.01)			
			95% CI	57.46-64.30		58.18-67.43		53.56-63.90			
		**9 months**									.35
			Mean (SD)	71.04 (12.31)		72.03 (12.71)		69.93 (11.86)			
			95% CI	68.81-73.28		68.83-75.23		66.75-73.11			
**Satisfaction**											
		**9 months**									.002^a^
			Mean (SD)	47.18 (4.46)		48.22 (3.86)		46.00 (4.82)			
			95% CI	46.37-47.99		47.25-49.20		44.71-47.29			

^a^Mann-Whitney *U* test (median, IQR): Mediterranean diet adherence at baseline (median 7.00, IQR 6.00-9.00); Mediterranean diet adherence at 9 months (median 12.00, IQR 9.00-13.00); nicotine dependence at 9 months (median 0.00, IQR 0.00-3.00); cardiovascular risk factor knowledge at 9 months (median 119.00, IQR 112.00-120.00); 12-item Short Form survey physical component score at 9 months (median 43.04, IQR 34.13-55.19); satisfaction (median 50, IQR 46.00-50.00).

^b^CVRF: cardiovascular risk factor.

^c^MGL MAQ: Morisky Green and Levine Medication Adherence Questionnaire.

^d^SF-12: 12-item Short Form survey.

^e^PCS: physical component score.

^f^MCS: mental component score.

^g^WHO-5: World Health Organization-5 Well-Being Index.

#### Smoking

Regarding stopping smoking, more participants did so in the mHealth group compared with those receiving standard health care (25/67, 75% vs 11/61, 42%; *P*=.01). The level of nicotine dependence also fell more in the mHealth group than in the control group (mean 0.93, SD 2.29 points vs mean 2.27, SD 2.82 points; *P*=.02; Table 2).

#### Adherence to the Mediterranean Diet and Frequency of Food Consumption

The score for adherence to the Mediterranean diet was significantly higher among the patients from the mHealth group (mean 11.83, SD 1.74 points) compared with the control group (mean 10.14, SD 2.02 points; *P*<.001) with an effect size (Cohen *d*) of 0.8. Moreover, the prevalence of adherence to the Mediterranean diet (score >9 points) was higher in the mHealth group than in the control group (57/67, 90% vs 42/61, 75%; *P*=.02). Regarding the frequency of food consumption, a significant reduction was observed in the consumption of red meat (≤1/week: 60/67, 95% vs 44/61, 77%; *P*=.001), and industrial pastries (<2/week: 60/67, 93% vs 52/61, 91%; *P*=.003) among the participants of the mHealth group compared with the control group. Furthermore, the patients in the mHealth group significantly increased their intake of vegetables (≥2/day: 48/67, 75% vs 28/61, 49%; *P*=.03), fruit (≥2/day: 58/67, 90% vs 39/61, 68%; *P*=.02), and whole-meal cereals (≥1/day: 41/67, 64% vs 20/61, 35%; *P*=.003).

#### Physical Activity

Likewise, the patients in the mHealth group spent significantly more time doing PA (patient-reported) every week than those in the control group (mean 619.14, SD 318.21 min/week vs mean 471.70, SD 261.43 min/week; *P*=.007), with an effect size (Cohen *d*) of 0.6.

#### Knowledge of CVRFs and a Healthy Lifestyle

The patients in the mHealth group presented a significantly higher level of knowledge about a healthy lifestyle and the control of CVRFs than those in the control group (mean 118.70, SD 2.65 points vs mean 111.25, SD 9.05 points; *P*<.001). Therapeutic adherence improved to a similar extent in both groups at the end of the follow-up period, with no significant differences being found between the groups.

#### Quality of Life and Well-Being

Regarding the quality of life, the physical component was significantly better among the patients in the intervention group (mean 45.80, SD 10.79 points vs mean 41.40, SD 10.78 points; *P*=.02), while both groups presented similar values for the mental component. As for the well-being index, the patients in the mHealth group presented slightly better scores, but they did not reach statistical significance.

#### Patient Overall Satisfaction

Regarding overall satisfaction with the health care received, the patients in the mHealth group scored significantly higher than those receiving standard health care, with respective average scores of 48.22 (SD 3.89) points and 46.00 (SD 4.82) points (*P*=.002).

### Secondary Outcomes

#### BMI, WC, SBP, DBP, Heart Rate, HbA1c, and Lipids

Regarding the secondary outcome variables (Multimedia Appendix 1), at the end of the follow-up period, lower values were obtained for BMI, WC, SBP, DBP, heart rate, total cholesterol, LDL cholesterol, HDL cholesterol, triglycerides, and HbA_1c_ in both the mHealth and control groups, with no significant differences being found.

#### Anxiety and Depression

Regarding emotional aspects, the levels of both anxiety and depression were lower at the end of the study than at the beginning, but no significant differences were observed between the groups after the follow-up.

#### MACE and Other Complications

Major adverse events (3/128, 2.4% of all the participants) and complications such as bleeding, angina, and coronary interventions on another vessel (9/128, 7.2% of all the participants) were scarce in both groups during the 9-month follow-up, with no significant differences being found between the intervention and control groups.

#### Patient App Satisfaction and Usability

The participants in the mHealth group gave high scores for both satisfaction with the app (mean 44.38, SD 6.176 points) and its usability (mean 95.22, SD 7.369 points).

## Discussion

### Main Results

This study of people with CAD after a PCI analyzed the effect of an educational intervention based on the use of an mHealth app on the following factors: changes in lifestyle in terms of diet, PA, and tobacco use; level of knowledge of a healthy lifestyle; and the control of CVRFs, therapeutic adherence, and quality of life. The results obtained show that the intervention led to significant improvements in lifestyle in terms of adherence to the Mediterranean diet, PA, and stopping smoking, and that the patients increased their knowledge about a healthy lifestyle and controlling CVRFs. The patients’ quality of life improved, and they reported a high level of satisfaction with the health care they received.

### Comparison With Previous Work

The Mediterranean diet is considered a cornerstone of a healthy lifestyle to prevent cardiovascular diseases [4,42,43]. In this study, adherence to the Mediterranean diet improved in both groups at the end of the study, but this improvement was more noticeable in the patients in the mHealth group. This group of patients also increased the frequency with which they consumed healthy foods, such as fruit, vegetables, and whole-meal cereals, and decreased the frequency with which they consumed unhealthy foods, such as red meat and industrial pastries, to a greater extent than those in the control group (Table 2). On the contrary, the clinical trial conducted by Choi et al [29] on the effectiveness of dietary advice provided to patients with heart conditions through a smartphone found that the mHealth intervention did not increase adherence to the Mediterranean diet. However, other authors, such as Widmer et al [44], reported a more favorable score for the consumption of healthy food among patients undergoing cardiac rehabilitation who used an mHealth app. Given our results, providing dietary guidance through a mobile app may be a practical way of achieving healthy changes in people’s diets, with the added advantage of using fewer resources than face-to-face visits.

As our results show, the time spent performing PA (patient-reported) was significantly higher in the mHealth group than in the control group. These results are in line with other clinical trials that observed improvements in the PA performed associated with an mHealth intervention [45-47]. However, Johnston et al [48] Did not find significant increases in the PA performed by patients using a health app for smartphones. Nevertheless, a recent meta-analysis has highlighted that mHealth apps are effective at encouraging patients to increase the amount of PA they do after a coronary event [18]. In the secondary prevention of CAD, one of the essential lifestyle changes that patients have to make is to perform regular PA, such as walking for at least 30 minutes every day [4,43]. For this reason, our results show that the mobile app was able to encourage the participants to start and continue doing more PA. However, our study did not have data available from an accelerometer as the PA was self-reported.

This study found an important decrease in the proportion of smokers and nicotine dependence measured by the Fagerström test at the end of the follow-up period in both the mHealth and control groups. However, the decrease in this addictive behavior was significantly greater among the participants in the mHealth group. Moreover, 75% (25/67) of the smokers in the mHealth group stopped smoking, while in the group receiving standard health care, only 42% (11/61) did so, even though at the beginning of the study the prevalence of smokers in the 2 groups was similar. Several meta-analyses have studied the effect of telehealth interventions on the prevalence of smoking, obtaining mixed results. While some [18,49] did not find statistically significant improvements in tobacco use when these technologies were used, another meta-analysis did observe a beneficial effect on stopping smoking among patients with CAD through the use of mHealth strategies [50]. Our positive results about stopping smoking with the use of the digital intervention could be due to several reasons, such as the support perceived by the patients and the interaction with the app that kept them motivated to change.

Our results show a significant improvement in the level of knowledge of a healthy lifestyle and the control of CVRFs among the patients using the health app compared to the control group. Few clinical trials involving mHealth interventions analyze the effectiveness of these interventions in improving the patient’s knowledge. Among these, Dorje et al [51] reported an increase in the level of knowledge among patients using a telehealth app. Furthermore, the results of the studies included in EUROASPIRE II highlighted that in patients who had experienced a coronary event, a correlation existed between knowledge of CAD and changes in lifestyle and therapeutic adherence [52]. Knowledge of the risk factors for the disease is an essential requirement for patients to decide to adopt behaviors in line with a healthy lifestyle. However, people also need to be motivated to incorporate such behavior into their daily lives [53-55]. Innovative mHealth technology could help to achieve both objectives by increasing the patients’ knowledge and motivation.

Therapeutic adherence improved to a similar extent at the end of the follow-up period in both groups, mHealth (54/67, 84%) and standard health care (44/61, 78%). Although data from previous studies indicate that adherence to treatment decreases progressively after a coronary event, reaching around 50% a year after experiencing a myocardial infarction [56-58], our results do not seem to support these data since the therapeutic adherence in both groups was greater than the figures reported by other authors. In addition, recent meta-analyses [18,59] have observed improvements in adherence to treatment in patients with cardiovascular disease with the use of mHealth apps with medication reminders or alarms on mobile devices.

Our results show that the patients in the mHealth group benefited from significant improvements in the physical component of quality of life compared with the participants receiving standard health care. These results are in partial agreement with a recent meta-analysis [18] but not with another meta-analysis [49] that did not observe significant increases in the quality of life of these patients. The favorable effects of the mHealth intervention highlighted by our results may partly be related to the remote health care option provided by this intervention, whereby patients could resolve queries without having to leave their homes. Patients were given support, thus enhancing their self-sufficiency and the perception they have of their health. Improvements in quality of life may, in turn, motivate patients to comply with secondary prevention recommendations.

The overall satisfaction with the health care received was significantly higher in the patients from the mHealth group than in the control group, and satisfaction with the mHealth app was also high (mean 44.38, SD 6.176 points out of 50). The average score for the usability of the app was 95.22 (SD 7.36) out of 100. These results agree with those obtained by Johnston et al [48]. Our positive results could be attributed to the fact that the patients perceived the app to be a support tool that provided them with information about their disease as well as the motivation to adopt a healthy lifestyle. Telehealth interventions have the potential to overcome barriers and could represent a ground-breaking alternative for providing cardiac rehabilitation to patients for whom face-to-face health care is more complicated. Factors to be taken into consideration when choosing this option could be the patient’s risk profile, preferences, and accessibility to health services.

The EVITE interactive tool provided relevant information about the disease, increased the patient’s knowledge of their health status, promoted the self-reporting of important CVRF variables, and supplied the patients with visual feedback about changes, motivating them to improve their health. The patients felt more connected with the health professionals, obviating the need for unnecessary visits for trivial reasons. Most of the patients in the mHealth group stated that they would like to continue using the interactive support tool and that they would recommend the tool to other patients.

Our results showed no significant differences between the 2 groups with regard to BMI and WC at the end of the study, although recent meta-analyses show controversial results [18,60].

Lipid variables, blood pressure, and HbA_1c_ did not show significant differences between the 2 groups. These results agree with recent meta-analyses [18,61], which could be due to the intensive prescription of secondary prevention medication among the participants.

Patients have been found to experience the symptoms of anxiety and depression straight after undergoing a PCI, and these may persist for months or even years [62,63]. However, our results show a decrease in the level of anxiety and depression during the follow-up period in both the mHealth group and the control group (Multimedia Appendix 1). These results are in line with previous studies [18], although not with another meta-analysis [60].

Major adverse events were very rare in both groups, as were hospital readmissions. As a result of the important improvements achieved in recent years in the treatment of patients with CAD, the prevalence of short-term adverse events is generally low in different trials.

### Clinically Meaningful Improvements in Primary Outcomes

The improvements observed in the mHealth group are clinically relevant, according to scientific evidence [4]. One year after myocardial infarction, patients who continue to smoke have worse cardiovascular outcomes and death than nonsmokers, and the long-term outcomes among those who quit smoking are comparable with those of nonsmokers [64]. We observed a 33% higher prevalence of smoking cessation in the mHealth group than in the control group.

Adults are recommended to perform at least 150-300 minutes a week of moderate-intensity PA, and additional benefits are gained with even more [4,65]. In our study, cardiologists advised the patients to walk for at least 1 hour every day, resulting in 420 minutes per week of PA. While all the participants in our study complied with the PA recommendations, those in the mHealth group performed more, so they would be expected to benefit from this because they are more active than the control group, with an important effect size (Cohen *d*) of 0.6. It should be noted that this study was performed in a geographical area in which the weather is generally good, which encourages people to go outside for a walk.

Adherence to the Mediterranean diet improves survival rates and reduces the risk of cardiovascular disease in individuals with CVD [66]. Moreover, greater adherence to this diet is associated with a 10% reduction in cardiovascular events or mortality [67]. In our study, the prevalence of good adherence to the Mediterranean diet (>9 points) was 15% higher among participants in the mHealth group. A meta-analysis reported a 4% lower risk of cardiovascular mortality for each additional serving of fruit and vegetables per day [68]. Likewise, a high intake of processed meat and unprocessed red meat is associated with an increased risk of atherosclerotic cardiovascular disease of 7% and 3%, respectively [69]. In our study, the prevalence of red meat consumption of ≤1 time per week was 18% higher in the mHealth group.

Knowledge of cardiovascular disease helps patients control risk factors and adopt behaviors that promote cardiovascular health [53,54]. For this reason, it could be considered that the more favorable results in the level of knowledge observed in our patients in the mHealth group could have a clinical impact.

Improving quality of life is one of the most important objectives to be achieved with CAD [70]. Thus, assessing quality of life allows the impact of the disease and its treatment on daily life to be determined. In our study, the score in the physical dimension of quality of life was statistically higher in the mHealth group, a finding that may be related to the capacity of the EVITE app to provide support to patients.

### Limitations and Strengths

Our study has a few limitations. First, the participants included in the mHealth group agreed to participate voluntarily and gave their written consent, meaning that they may have been more motivated to adhere to secondary prevention. A second limitation could be that to participate in the mHealth programs, the patients had to have a mobile phone or tablet with an internet connection, which could suggest that the participants were younger. However, 99% of the European population currently has a mobile telephone, and 86% of homes have an internet connection [71], although our results could also be due to the fact that participants in the mHealth group had a higher socioeconomic status. Moreover, women were less represented in our study, as only 29% (36/128 of the participants belonged to this population group. The third limitation is the limited sample size and the duration of the follow-up. The barrier to recruiting more participants was that the hospital was occupied by patients with COVID-19. A larger sample size and a longer follow-up could enhance the knowledge gleaned about the efficacy of the intervention. The fourth limitation is that most of the primary outcomes were self-reported and the study was open-label, but we assumed the patients responded honestly since they filled out the questionnaire without indicating their names. The questionnaires only contained an identification number so that the statisticians could record information in the database. This was explained to the patients so that they could respond freely. The fifth limitation is detection and performance bias since the blinding of the participants was not possible because of the nature of the intervention. The coordinators and the researchers analyzing the results were blinded to the allocation of participants, however. Finally, this study was conducted at a single public specialty reference hospital, and it would be necessary to test whether the favorable effects observed are generalizable to other health care settings. Additionally, in our study, we did not carry out an economic evaluation that could provide useful information to clarify whether the implementation of this mHealth intervention in the health care of these patients is cost-effective.

This study also presents some strengths, such as the fact that the sample is representative of patients who experience a coronary event in different localities, as the study was conducted on patients treated in the cardiology unit of a reference hospital that receives patients from both rural and urban areas. In addition, all the instruments and tools used in the clinical trial were validated internationally, which allows for generalization and for the study to be compared with others. Another strength was that the trial was conducted using a smartphone app, so the intervention involved the latest, up-to-date technology. In addition, the study included exclusively patients with CAD, so the sample of patients was more homogeneous than in studies that included patients with cardiovascular disease. The inclusion of different kinds of behavioral, metabolic, and psychosocial variables provides a broad view of the results obtained with mHealth technology. Finally, most previous trials were conducted with apps that encouraged behavioral changes related to performing PA, weight loss, or therapeutic adherence but did not comprehensively tackle all the CVRFs as our app does.

### Conclusions

Using the EVITE app with patients who have experienced a coronary event is an effective way to improve their lifestyle in terms of adherence to the Mediterranean diet, frequency of healthy food consumption, PA, stopping smoking, knowledge of a healthy lifestyle, and controlling CVRFs, quality of life, and satisfaction with the health care received.

More studies are required to examine the impact of smartphone interventions on people who have undergone a coronary event, with long-term follow-ups that analyze mortality and cardiac-cause hospitalization, because these are important yardsticks of the success of secondary prevention strategies that make it possible to establish the clinical importance of the findings. Cost analyses are also required to promote the generalized use of these tools, their implementation, and their feasibility.

## Appendix

Multimedia Appendix 1 Secondary outcome variables at baseline and 9 months.

Multimedia Appendix 2 CONSORT-eHEALTH checklist (V 1.6.1).

## Abbreviations

CAD: coronary artery disease
CVRF: cardiovascular risk factor
DBP: diastolic blood pressure
HbA1c: glycated hemoglobin
HDL: high-density lipoprotein
LDL: low-density lipoprotein
MACE: major adverse cardiovascular event
MET: metabolic equivalent
MGL MAQ: Morisky Green and Levine Medication Adherence Questionnaire
mHealth: mobile health
PA: physical activity
PCI: percutaneous coronary intervention
SBP: systolic blood pressure
SF-12: 12-item Short Form survey
SUS: System Usability Scale
WC: waist circumference
