# Supplementary material for: Effectiveness of an Interactive mHealth App (EVITE) in Improving Lifestyle After a Coronary Event: Randomized Controlled Trial
Source: JMIR Mhealth Uhealth. 2024 Apr 22;12:e48756. doi: 10.2196/48756 (PMC11074898; doi:10.2196/48756)
Supplement: Multimedia Appendix 1 [file mhealth_v12i1e48756_app1.doc]

**Multimedia Appendix 1. Secondary outcome variables at baseline and 9 months.**

|  |  |  | | Total (N=128) | mHealth (n=67) | Control (n=61) | *P* value |
| --- | --- | --- | --- | --- | --- | --- | --- |
| **BMI (Kg/m2), mean±DE (IC95%)** | | | |  |  |  |  |
|  |  | 9 months | | 28.10±4.97  (27.19-29.01) | 27.92±5.14  (26.64-29.21) | 28.30±4.81  (26.99-29.62) | 0.68 |
| **WC (cm), mean±DE (IC95%)** | | | |  |  |  |  |
|  |  | 9 months | | 100.5±12.09  (96.69-104.33) | 98.73±12.37  (93.73-103.73) | 103.60±11.33  (97.32-109.88) | 0.21 |
| **SBP (mmHg), mean±DE (IC95%)** | | | |  |  |  |  |
|  |  | Baseline | | 132.01±19.77  (128.55-135.47) | 132.42±20.97  (127.30-137.53) | 131.56±18.53  (126.81-136.31) | 0.80 |
|  |  | 9 months | | 123.97±11.71  (121.85-126.10) | 123.18±11.86  (120.26-126.10) | 124.96±11.55  (121.78-128.15) | 0.41 |
| **DBP (mmHg), mean±DE (IC95%)** | | | |  |  |  |  |
|  |  | Baseline | | 75.17±11.12  (73.23-77.12) | 77.01±11.54  (74.20-79.83) | 73.15±10.37  (70.49-75.80) | 0.04 |
|  |  | 9 months | | 69.42±10.68  (67.50-71.34) | 69.85±8.61  (67.73-71.97) | 68.89±12.72  (65.41-72.36) | 0.62 |
| **HR (beats/min), mean±DE (IC95%)** | | | |  |  |  |  |
|  |  | Baseline | | 72.92±12.47  (70.74-75.10) | 73.21±11.88  (70.31-76.11) | 72.61±13.17  (69.23-75.98) | 0.78 |
|  |  | 9 months | | 63.64±8.63  (62.06-65.21) | 64.02±9.00  (61.80-66.23) | 63.15±8.20  (60.87-65.44) | 0.59 |
| **HbA1C (%), mean±DE (IC95%)** | | | |  |  |  |  |
|  |  | Baseline | | 6.50±1.61  (6.17-6.84) | 6.12±1.18  (5.78-6.46) | 6.97±1.93  (6.36-7.58) | 0.02^a^ |
|  |  | 9 months | | 6.05±1.38  (5.69-6.42) | 5.78±1.40  (5.28-6.27) | 6.44±1.29  (5.89-6.98) | 0.07 |
| **TC (mg/dl), mean±DE (IC95%)** | | | |  |  |  |  |
|  |  | Baseline | | 184.19±50.52  (175.17-193.20) | 190.28±46.03  (178.87-201.68) | 177.36±54.72  (162.97-191.75) | 0.15 |
|  |  | 9 months | | 121.27±30.65  (115.63-126.91) | 118.43±25.13  (111.99-124.86) | 124.42±35.77  (114.75-134.09) | 0.29 |
| **HDL-C (mg/dl), mean±DE (IC95%)** | | | |  |  |  |  |
|  |  | Baseline | | 40.55±11.61  (38.46-42.64) | 40.98±10.41  (38.40-43.57) | 40.05±12.95  (36.59-43.52) | 0.66 |
|  |  | 9 months | | 43.23±11.95  (40.98-45.47) | 43.12±9.29  (40.70-45.54) | 43.35±14.49  (39.31-47.38) | 0.92 |
| **LDL-C (mg/dl), mean±DE (IC95%)** | | | |  |  |  |  |
|  |  | Baseline | | 117.25±42.00  (109.72-124.77) | 124.03±41.39  (113.77-134.29) | 109.51±41.69  (98.45-120.57) | 0.056 |
|  |  | 9 months | | 59.86±36.37  (53.05-66.67) | 56.03±20.22  (50.76-61.30) | 64.11±48.30  (50.80-77.43) | 0.24 |
| **TG (mg/dl), mean±DE (IC95%)** | | | |  |  |  |  |
|  |  | Baseline | | 159.14±93.97  (142.16-176.13) | 151.55±77.14  (133.28-170.82) | 167.82±110.20  (138.31-197.33) | 0.34 |
|  |  | 9 months | | 113.85±58.54  (103.09-124.62) | 105.90±53.19  (92.28-119.53) | 122.67±63.27  (105.57-139.78) | 0.12 |
| **GOLDBERG** | | | |  |  |  |  |
|  | **Anxiety (Score), mean±DE (IC95%)** | | |  |  |  |  |
|  |  | *Baseline* | | 3.27±3.07  (2.74-3.81) | 3.30±3.04  (2.56-4.04) | 3.25±3.13  (2.44-4.05) | 0.92 |
|  |  | *9 months* | | 2.36±2.91  (1.83-2.90) | 2.50±3.07  (1.72-3.28) | 2.21±2.74  (1.48-2.95) | 0.59 |
|  | **Depression (Score ), mean±DE (IC95%)** | | |  |  |  |  |
|  |  | | *Baseline* | 2.13±2.75  (1.65-2.61) | 1.91±2.60  (1.27-2.54) | 2.37±2.90  (1.63-3.12) | 0.34 |
|  |  | | *9 months* | 1.11±2.07  (0.731-1.48) | 1.09±2.14  (0.55-1.64) | 1.12±2.01  (0.58-1.66) | 0.94 |
| **MACCE, n (%)** | | | |  |  |  |  |
|  |  | | 9 months | 3 (2.4) | 1 (1.5) | 2 (3.3) | 0.49 |
| **OTHER COMPLICATIONS ^b^, n (%)** | | | |  |  |  |  |
|  |  | | 9 months | 9 (7.2) | 6(9.2) | 3 (5.0) | 0.36 |
| **Satisfacction app (score), mean±DE (IC95%)** | | | |  |  |  |  |
|  |  | | 9 months | - | 44.38±6.176 | - | - |
| **Usability (score), mean±DE (IC95%)** | | | |  |  |  |  |
|  |  | | 9 months | - | 95.22±7.369 | - | - |

^a^ U-Mann Whitney [Median (IQR)]: HbA1c Baseline [5.90 (6.90-5.50)].

^b^Other complications: Bleeding, Angina, ReACTP other vessel App: application; BMI: body mass index; CLDL: high-density lipoprotein cholesterol; DBP: diastolic blood pressure; HbA1c: glycosylated hemoglobin; HDL-C: low-density lipoprotein cholesterol; HR: heart rate; MACCE: major cardiovascular and cerebrovascular adverse events; SBP: systolic blood pressure; TC: total cholesterol; TG: triglycerides.

App: application; BMI: body mass index; CLDL: high-density lipoprotein cholesterol; DBP: diastolic blood pressure; HbA1c: glycosylated hemoglobin; HDL-C: low-density lipoprotein cholesterol; HR: heart rate; MACCE: major cardiovascular and cerebrovascular adverse events; SBP: systolic blood pressure; TC: total cholesterol; TG: triglycerides.
